# Enhancing the metabolic benefits of exercise: Is timing the key?

**DOI:** 10.3389/fendo.2023.987208

**Published:** 2023-02-15

**Authors:** Samuel Bennett, Shogo Sato

**Affiliations:** Center for Biological Clocks Research, Department of Biology, Texas A&M University, College Station, TX, United States

**Keywords:** exercise, cardiometabolic health, metabolic diseases, circadian biology, time-of-exercise, metabolism, physical activity, high-throughput omics data

## Abstract

Physical activity represents a potent, non-pharmacological intervention delaying the onset of over 40 chronic metabolic and cardiovascular diseases, including type 2 diabetes, coronary heart disease, and reducing all-cause mortality. Acute exercise improves glucose homeostasis, with regular participation in physical activity promoting long-term improvements in insulin sensitivity spanning healthy and disease population groups. At the skeletal muscle level, exercise promotes significant cellular reprogramming of metabolic pathways through the activation of mechano- and metabolic sensors, which coordinate downstream activation of transcription factors, augmenting target gene transcription associated with substrate metabolism and mitochondrial biogenesis. It is well established that frequency, intensity, duration, and modality of exercise play a critical role in the type and magnitude of adaptation; albeit, exercise is increasingly considered a vital lifestyle factor with a critical role in the entrainment of the biological clock. Recent research efforts revealed the time-of-day-dependent impact of exercise on metabolism, adaptation, performance, and subsequent health outcomes. The synchrony between external environmental and behavioural cues with internal molecular circadian clock activity is a crucial regulator of circadian homeostasis in physiology and metabolism, defining distinct metabolic and physiological responses to exercise unique to the time of day. Optimising exercise outcomes following when to exercise would be essential to establishing personalised exercise medicine depending on exercise objectives linked to disease states. We aim to provide an overview of the bimodal impact of exercise timing, i.e. the role of exercise as a time-giver (*zeitgeber*) to improve circadian clock alignment and the underpinning clock control of metabolism and the temporal impact of exercise timing on the metabolic and functional outcomes associated with exercise. We will propose research opportunities that may further our understanding of the metabolic rewiring induced by specific exercise timing.

## 1 Introduction

The circadian clock is an essential evolutionary mechanism permitting biological functions to be temporally controlled and pre-emptively coordinated in anticipation of food intake and physical activity. Located in the hypothalamic suprachiasmatic nucleus (SCN) of the brain, the central circadian clock or ‘master pacemaker’ is entrained by light and synchronises peripheral molecular clocks at the tissue level (1). The central and peripheral clocks ensure 24-hour rhythms of biological functions, including sleep-wake cycles, hormone secretion, cardiovascular function and whole-body metabolism (2). Although core clock machinery is ubiquitous across all tissues and cell types, clock transcriptional output is highly tissue-specific and is impacted by physiological cues such as eating, stress and physical activity (3); as such, the circadian clock induces 24-hour oscillations in substrate metabolism, glucose homeostasis (4), skeletal muscle insulin sensitivity (5) and oxidative capacity (6).

Circadian entrainment that aligns internal molecular clock rhythms with external time cues, such as waking and feeding times, is critical to maintaining circadian biological homeostasis. Thus, misalignment between the internal molecular clock and timed environmental cues is associated with several adverse health outcomes, as evidenced by large-scale epidemiological studies. For example, robust relationships between shift work, specifically night shifts, and metabolic disease risk with data from the Nurse Health Study revealing an increased risk of ischaemic stroke (7) and diabetes (8) in night-shift working participants compared to day working colleagues. In addition, Scandinavian population studies support the relationship between sleep disturbance and metabolic health, with Swedish and Danish night shift workers exhibiting greater obesity risk, lipid profile abnormalities, impaired glucose tolerance and diabetes compared to day-working colleagues (9, 10). Whilst correlation does not necessarily equal causality, it must be noted that simulated shift work experiments have revealed transcriptomic (11), endocrine (12, 13) and metabolic (14) circadian misalignment following relatively short intervention periods.

Shift working aside; the modern human lifestyle poses a severe threat to the entrainment and coordination of central and peripheral clocks with artificial 24-hour lighting, prolonged periods of inactivity and access to energy-dense foods, all disrupting the circadian rhythm (15–17). Notably, a recent cross-sectional investigation utilising primary skeletal muscle cells from type-2 diabetic and healthy control participants revealed divergent skeletal muscle mitochondrial metabolism (18) and altered clock-controlled gene expression, leading to impaired mitochondrial respiration in T2D skeletal muscle. Accordingly, exercise represents a significant, non-pharmacological intervention capable of preventing or delaying the onset of metabolic disease and reversing the adverse effects of the modern human lifestyle, including physical inactivity and poor diet (19, 20).

The timing of exercise has profound effects on physical capacity, homeostatic disturbance, and metabolic responses, potentially providing two-fold benefits. Firstly, exercise timing may act as a crucial time-giver (*zeitgeber*) for individuals and has been proposed as a strategy to synchronise and realign disrupted central and peripheral circadian clocks (21). Secondly, the circadian regulation of exercise, including the diurnal variation in exercise performance (22, 23) and alterations to substrate metabolism (17, 24, 25), may potentiate the beneficial effects of exercise and subsequent health outcomes.

On this basis, the present narrative review aims to provide an overview of the bimodal impact of exercise timing, i.e., the role of exercise as a *zeitgeber* to improve circadian clock alignment and the temporal impact of exercise timing on the metabolic and functional outcomes associated with exercise. We will highlight research opportunities that may identify exercise “prime times” for the biological outcomes and health benefits in response to exercise.

## 2 Exercise as a *Zeitgeber*

First proposed in 1951 by Jurgen Aschoff, the term *zeitgeber* (From the German for “time-giver”) (26) was described as an external stimulus that synchronises biological functions across a 24-hour cycle (27). Light is a critical *zeitgeber* that entrains the central circadian clock housed in hypothalamic suprachiasmatic nuclear (SCN) and synchronises peripheral tissue clocks (1) *via* neuroendocrine mechanisms (28, 29). The role of exercise as a potential *zeitgeber* has been discussed in the systematic review by Lewis and Colleagues (30), highlighting the impact of exercise on circadian phase shifts, the diurnal variation in exercise performance and the interaction between exercise and the circadian system to improve health.

Investigating the role of exercise as a *zeitgeber* is inherently difficult. It requires the precise control of both photic (light, either solar or ambient) and non-photic *zeitgebers*, including time of feeding and physical activity (31). Whilst the direct measurement of the molecular circadian clock activity is inherently problematic in humans, exercise has been repeatedly reported to induce significant phase shifts *via* several proxies of circadian rhythm, including hormone secretion (e.g., melatonin, cortisol and thyroid stimulating hormone [TSH]) and physiological parameters (e.g., body temperature and blood pressure) (32–38). Firstly, exercise can be introduced into ‘real-life’ situations of circadian misalignment to induce a circadian phase shift and realign circadian rhythms, albeit this approach is hampered by the difficulty in removing confounding variables. Attempting to alleviate the symptoms of jet lag and improve circadian alignment following trans-Atlantic travel, Milan-based endurance runners who completed daily evening exercise pre-travel observed improved sleep and circadian rest-activity cycles on arrival in New York (39). Despite reporting that bedtimes and wake times were controlled for each participant, the specific times were unclear in the study. As a result, altered light exposure may have contributed to improved adaptation masking the *zeitgeber* effect of exercise.

Alternatively, a “constant routine” approach may be implemented whereby sleep-wake cycles, mealtimes and physical activity are standardised before, during and after the introduction of the *zeitgeber* in question (i.e. exercise). By utilising this approach, any alteration in circadian rhythm may be attributed to introducing the ‘new’ *zeitgeber*, albeit with diminished ecological validity compared to the previous approach. Van Reeth et al. (36 *)*, were amongst the first to implement a constant routine approach to investigate the circadian phase-shifting effects of nocturnal exercise. To control for potential confounding zeitgebers, participants adhered to a constant routine for 7 days before each experimental trial, and food intake was prohibited. Instead, a constant intravenous infusion of glucose (5·g·kg·day^-1^) provided all caloric intake.

Additionally, exercise was standardised to the core body temperature nadir with the midpoint of exercise timed to 3 hours before, at and 2 hours after minimum core body temperature. Ultimately, night-time exercise was associated with melatonin and thyroid-stimulating hormone phase delays, with delays tending to be smaller when exercise was completed in the latter part of the night-time/early morning. Constant routine approaches have been implemented with varying levels of control, with some controlling for sleep-wake cycles, physical activity and nutritional intake with glucose infusion (33) or controlled mealtimes (32, 37) and have all shown robust melatonin phase delays when exercise is completed in the evening or overnight.

The mechanism by which exercise induces these phase shifts remains unclear. Nevertheless, the fact that repeated bouts of exercise stimulus were required to induce melatonin phase shifts suggests a *zeitgeber* effect of exercise was present (32). Specifically, morning exercise results in melatonin secretion phase delays whilst evening exercise-induced phase advances (33). The counter-intuitive phase shifting reported in response to exercise relative to light exposure adds to the notion that exercise *per se* induced the phase shift rather than light exposure.

Based on the current evidence available, it is appropriate to conclude that exercise has phase-shifting capabilities and based upon physiological changes in body temperature (38), melatonin (40) and thyroid stimulating hormone secretion (36) highlight exercise’s capacity to synchronise circadian rhythms. Furthermore, the entrainment of the biological clock may occur through exercise-induced changes in AMPK (41), transcriptional coactivator PGC-1α (42) and the transcription factor HIF-1α (43) activities, all of which have been associated with the molecular clock. Previous work from our group has highlighted the direct effect of exercise on the biological clock in mice (24) with supporting data in humans following both endurance (44) and resistance type exercise (45). Whilst the relationship between exercise and the biological clock is increasingly accepted, whether an optimal time exists to complete exercise remains to be resolved.

## 3 Exercise timing improves lifestyle factors and health

Whilst the benefits of exercise for health are beyond the scope of this review, the appreciation of physical activity as a key lifestyle factor for healthy living is well established (46–48). Nevertheless, the holistic benefits of exercise and the potential to correct misaligned circadian rhythms in at-risk populations present a unique opportunity to optimise exercise prescription (37). For instance, timed exercise may enhance the circadian rhythm entrainment in shift working individuals, potentially restoring temporal hormone secretion such as melatonin (33), improving sleep homeostasis and reducing the negative impact of health implications of the disturbance of sleep/wake cycles (11–13). When discussing the impact of timed exercise on circadian clock function and health, it is essential to consider the diurnal variation in exercise capacity (49). Improved exercise capacity coincides with several circadian-controlled biological functions collectively contributing to improved exercise capacity. Firstly, core body temperature is greatest during the late afternoon/early evening; unsurprisingly, skeletal muscle strength and oxidative capacity show comparable time-of-day variations and typically peak during the late afternoon and early evening, respectively (6, 50, 51). Furthermore, systemic hormone and metabolite concentrations show distinct responses to different times of exercise (24, 52, 53), which may contribute to time-specific exercise outcomes (54, 55).

The impact of exercise timing on diurnal exercise performance is a popular strategy for investigating the crosstalk between physical activity and circadian rhythms, with multiple studies utilising this approach (55–62). However, it is important to note that a ubiquitous limitation of this literature is the inability to isolate the impact of timed exercise relative to other *zeitgebers*, such as light exposure or mealtimes. Principally, morning exercise groups may wake and eat earlier, altering participants’ regular sleep-wake cycles and meal schedules, and may result in performance improvements due to phase advances. However, regarding performance differences, several studies have also reported changes in core temperature, with no difference in the circadian oscillation of core temperature between morning and evening exercise groups (60, 62). Additionally, no change in testosterone or cortisol was observed following 12 weeks of morning or evening exercise (55), raising the question of why changes in performance occur in the absence of changes in physiological markers of circadian rhythm.

Several studies highlight the interaction between exercise and the circadian system to improve health outcomes. As early as 1997, Van Someren and Colleagues (63) reported that 3 months of routine aerobic exercise, completed 3 times weekly, counteracted age-related fragmentation of the circadian clock, resulting in improved sleep quality in otherwise healthy older men (73 ± 2 years). Following the intervention, maximal aerobic fitness (VO_2max_) was significantly correlated with lower intraday variability in circadian rhythm, a critical finding given the association between aerobic capacity and all-cause mortality (46, 47). As well as improving sleep variables in older men, individualised exercise prescription improved sleep in patients with lung cancer compared to non-exercise controls (64), offsetting dysfunctional rest-activity rhythms pre-intervention (65). Notably, the benefit of prescribed exercise was greatest in individuals with poorer rest-activity rhythms, suggesting that the improvements observed may have had an underlying circadian component.

Human evidence highlighting the impact of exercise timing on health outcomes remains limited, with much of our current appreciation for the role of the circadian clock function and health inferred from animal models and epidemiological studies. However, recent evidence has provided crucial insight into the potential for timed exercise to improve metabolic health, with afternoon exercise revealing greater efficacy for improving glucose control in type 2 diabetes patients compared to morning-trained participants (66). Further multi-omics, multi-tissue (blood plasma, skeletal muscle, and adipose tissue) investigations from the same research group revealed significant temporal specificity following morning and evening exercise (67). Specifically, plasma carbohydrates were increased, with a decrease in skeletal muscle lipids following morning exercise, whereas afternoon HIT increased skeletal muscle lipids and mitochondrial protein content to a greater degree than morning training. Whilst the clinical significance of these findings warrants further investigation, the data presented by these authors support the notion that precisely timed exercise may lead to different exercise outcomes.

## 4 Omics approaches to capture metabolic signatures unique to exercise timing

The robust molecular interaction of the circadian clock to metabolic response to exercise indicates that exercise’s metabolic outputs might depend on its daily timing (17). Previous literature supports this notion that the timing of exercise, in addition to the quantity, duration, and type of exercise, could be one of the variables for metabolic and physiological responses to exercise (68, 69). However, understanding how the time of exercise rewires metabolic pathways in skeletal muscle and changes systemic metabolic adaptation remains an outstanding question. ‘Omics’ approaches, including metabolomics, transcriptomics, and proteomics, are considered effective in dissecting exercise’s time-of-day-dependent impact across the molecular time course of adaptation. Primarily global metabolite profiling provides a real-time fingerprint of metabolic activity and allows the identification of novel biochemicals unique to exercise timing. These may prove to be crucial diagnostic markers of metabolic disease patients and underpin differing metabolic responses to timed exercise.

**Figure 1:**
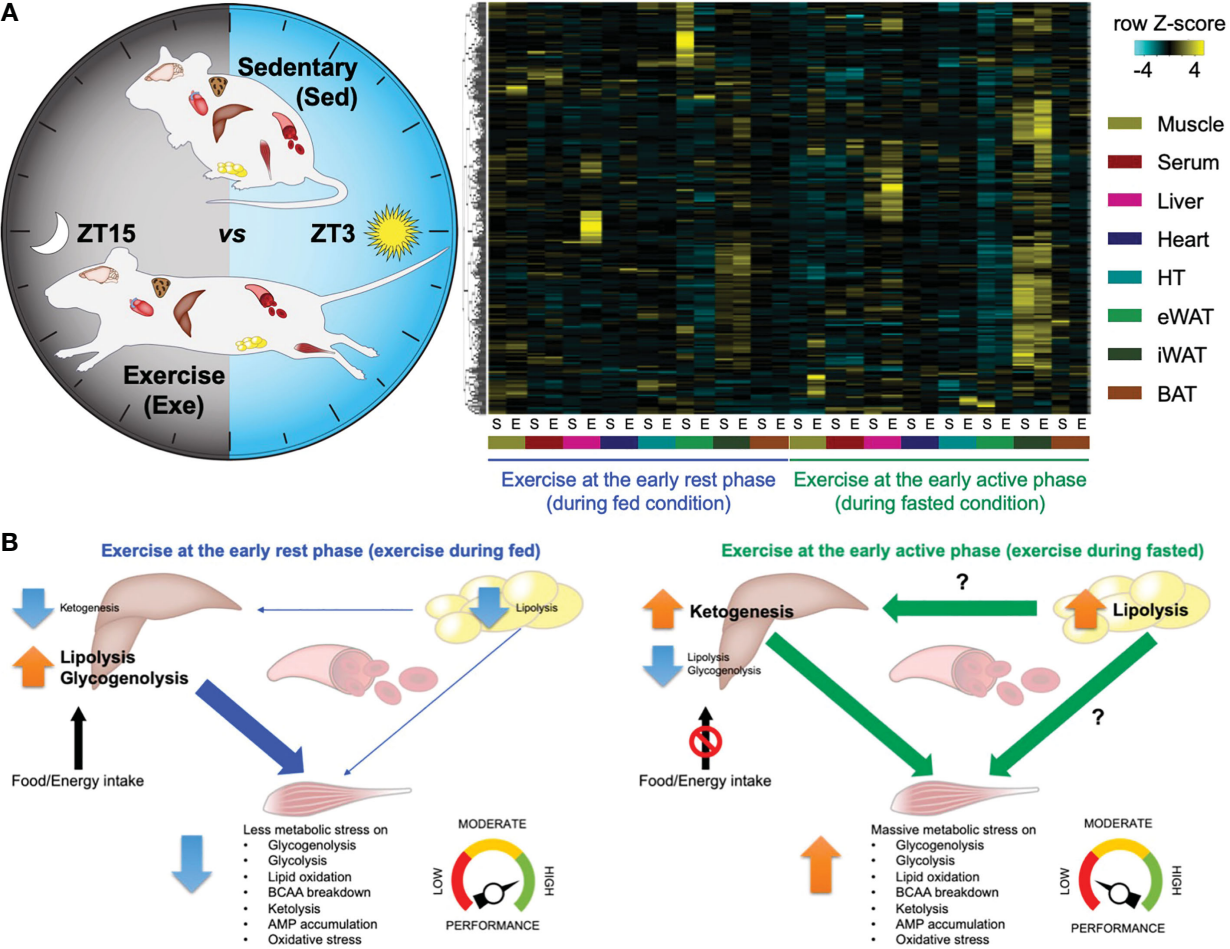
– Atlas of exercise metabolism unique to the time of day reveals tissue-dependent and collective metabolic responses to timed exercise (52). **(A)** Multi-tissue metabolomics upon exercise at the early rest (ZT3) versus the early active phase (ZT15). Heatmap displaying metabolites significantly changed only in a specific tissue and only after specific timing of exercise. **(B)** Schematic summary representing time-of-exercise-specificity of types of energy and the specific tissue of origin for energy during exercise. This figure represents a graphical representation of metabolomics data generated with the author’s permission and data from Sato et al., 2022 (52).

Previously, our group performed high-throughput transcriptome and metabolome analyses in mouse skeletal muscle in response to a single-bout treadmill running exercise at different times of the day (24). Significantly, many transcripts and metabolites are intensively changed only upon exercise at the early active phase (biological morning) rather than the early rest phase (biological night), suggesting a substantial impact on skeletal muscle metabolic pathways. Performing integrative analysis of the transcriptome and metabolome dataset, we revealed that exercise in the morning selectively rewires skeletal muscle energy metabolic pathways, including glycolytic pathways, lipid oxidation, amino acid breakdown, and ketone metabolism. This skeletal muscle multi-omics study uncovers a functional commitment of exercise timing to determine exercise energy utilisation in skeletal muscle. An accompanying study from Asher’s group also performed mouse skeletal muscle metabolomic profiling upon exercise at the early versus the late active phase, underscoring the time-of-day-dependent impact of exercise on energy metabolism followed by endurance exercise performance (70). The molecular circadian clock modulates the exercise timing-dependent metabolic response and exercise capacity (53). In addition, proteomic and phosphoproteomic profiles upon acute exercise at different daily timing also point to differential biological responses in skeletal muscle unique to the exercise timing (71). Proteins involved in energy provision and catabolic pathways are preferentially enriched after morning and evening exercise. Lastly, skeletal muscle and multiple organ systems dependently and collectively respond to exercise unique to the time of day (52). Our follow-up study investigated global metabolite profiles on 8 different tissues (skeletal muscle, liver, heart, white adipose tissues, brown adipose tissue, hypothalamus, and blood) collected from mice subjected to an acute treadmill exercise at different times of the day (**Figure 1A**). This allowed the detection of hundreds of different signalling molecules, of both metabolic and endocrine origin, across multiple tissues and to investigate their change in response to exercise at differing times of the day. Comparative analyses of cross-tissue metabolite dynamics, as well as an arteriovenous sampling of hindlimb muscle and sampling across the liver, lead to a deeper understanding of time-of-day-specific tissue cross-talk following exercise (**Figure 1B**). Finally, the study identified new exercise-induced signalling molecules (exerkines) in multiple tissues, which require further investigation to understand how they can individually or collectively influence health. The most recent study unveiled a comprehensive response of multi-tissue and multi-omics to exercise training at different times of the day in men with type 2 diabetes, revealing coordinated systemic metabolic adaptation to timed exercise (67). Thus, omics resources direct further research that can help us better understand how exercise, if timed correctly, can improve human health.

## 5 Research opportunities

Exercise-induced signalling metabolites (so-called exerkines) act as stimuli and substrates for cellular energy sensors and signalling molecules and induce adaptive rewiring of signalling pathways and transcriptional networks in response to exercise training (72). Novel investigations by our group have highlighted the impact of time-of-day of exercise on metabolic responses to acute exercise in skeletal muscle (24), with more recent work providing multi-tissue metabolomic analysis in response to time-of-day specific exercise (52). This work revealed exercise-stimulated signalling molecules capable of transducing metabolic information and mediating circadian reprogramming *via* energy sensors, AMP-activated protein kinase (AMPK), histone modifiers, e.g., histone deacetylases (HDACs) and transcription factors, including hypoxia-inducible factor 1α (HIF-1α). Alongside mechanisms for circadian reprogramming in response to exercise timing, work from our group highlights several metabolites of interest which have demonstrated capacity for clock modification through metabolic and epigenetic mechanisms. Whilst these metabolites have shown to be impactful in animal models, the translational applicability to humans remains to be seen. Future research should investigate whether these molecules’ administration may enhance the efficacy of timed exercise interventions or offer a potential pharmacological intervention for populations with misaligned circadian clocks to delay or prevent the onset of metabolic disease.

## 6 Conclusion

This review has discussed the bimodal impact of exercise timing on the circadian clock, including the realignment of misaligned clocks and the underpinning impact on metabolic regulation and health outcomes. First, it is essential to consider the population groups of interest within these studies. Crucially, individuals with metabolic disease risk are unlikely to be completing recommended daily/weekly physical activity; as such, the primary focus for this population should be promoting exercise participation, irrespective of timing to improve overall health. Secondly, exercise timing may only confer a trivial benefit relative to any exercise. Nevertheless, recent evidence has provided crucial insight into the metabolic impact of timed exercise in metabolically diseased humans, revealing robust differences between morning and evening exercise and providing early evidence to support the notion of “fine-tuning” exercise completion for the optimisation of health outcomes.

Whilst the timing of exercise may represent a novel strategy for optimising health outcomes and realignment of the circadian clock, it is essential to note that exercise at any time of day is of greater benefit than no exercise. In population groups who fail to meet daily recommended physical activity targets, the timing of exercise may offer trivial differences compared to exercise at any time.

## Author contributions

SB and SS wrote the manuscript, prepared the figure, and approved the final version. All authors contributed to the article and approved the submitted version.
